# Statins, skeletal muscle, and ryanodine receptor activation: resolving a 30-year mystery behind statin myotoxicity

**DOI:** 10.1186/s40842-025-00267-z

**Published:** 2025-12-23

**Authors:** Gaetano Santulli

**Affiliations:** Department of Molecular, Cellular and Biomedical Sciences, School of Medicine, City University of New York, Manhattan, NY 10031 USA

**Keywords:** Atorvastatin, Cryo-EM, Muscle toxicity, Ryanodine receptor, RyR1 activation, Statin intolerance, Statins, Structural biology

## Abstract

Statins are foundational therapies in cardiometabolic disease prevention, yet their clinical utility is could be limited by muscle-related adverse effects whose molecular origins have remained incompletely understood. In a landmark structural study, atorvastatin is shown to bind directly to skeletal muscle RyR1 in a unique triplet configuration, sequentially destabilizing the closed channel and promoting pathological Ca²⁺ leak. This work provides the first atomic-level explanation for statin-induced myopathy and reveals why individuals with RyR1 gain-of-function variants—already known to be overrepresented among statin-intolerant patients—are particularly vulnerable to muscle toxicity. For endocrinology, these findings are especially significant: statins remain central interventions in dyslipidemia, diabetes, obesity, and metabolic syndrome, and intolerance disproportionately affects individuals with high cardiometabolic burden. By establishing a receptor-mediated mechanism of statin myotoxicity, this study opens the door to genotype-informed statin prescribing, rational engineering of “RyR1-silent” lipid-lowering agents, and improved long-term adherence in patients with endocrine and metabolic disorders. These findings represent a major advance at the interface of metabolism, muscle physiology, and precision pharmacology, with the potential to reshape clinical practice across the endocrine continuum.

Statins have transformed cardiovascular medicine. Since their introduction, these inhibitors of 3-hydroxy-3-methyl-glutaryl-coenzyme A reductase (HMG-CoA reductase) have reduced major cardiovascular events on a global scale and currently rank among the most prescribed medications worldwide [1]. Yet, parallel to their monumental success is a paradox that has challenged clinicians and researchers for more than three decades: the incidence and wide heterogeneity of muscle-related adverse effects [2]. The clinical spectrum of statin-associated muscle symptoms spans from mild, cramp-like discomfort or myalgic complaints without elevations in creatine kinase (CK), to overt hyperCKemia with variable degrees of proximal limb-girdle weakness that can impair daily function. On the most severe end of this continuum are rare but potentially life-threatening presentations, including rhabdomyolysis and immune-mediated necrotizing myopathy (IMNM), the latter typically characterized by markedly elevated CK levels, progressive weakness, dysphagia, cardiopulmonary involvement, and positivity for anti-HMGCR antibodies, often requiring treatment with corticosteroids, immunosuppressive agents, and/or intravenous immunoglobulins [3, 4]. Despite extensive investigation, the molecular basis and predictors of such statin intolerance remain incompletely understood, with proposed mechanisms including mitochondrial dysfunction, impaired isoprenoid biosynthesis, disruptions in coenzyme Q10 homeostasis, and altered membrane cholesterol composition—each supported by partial but inconclusive evidence [5–10]. Into this space steps the landmark study led by Filip Van Petegem [11], which leverages high-resolution cryogenic electron microscopy (cryo-EM) to provide definitive evidence that atorvastatin binds directly to—and aberrantly activates—the skeletal muscle ryanodine receptor (RyR1). This work not only identifies a long-suspected culprit but also elucidates a structural mechanism that rewrites our understanding of statin myotoxicity.

## The long-suspected link between statins and RyR1

Ryanodine receptors control intracellular calcium release from the sarcoplasmic reticulum (SR), tightly regulating excitation–contraction coupling [12, 13]. Disturbances to RyR1 function—whether due to genetic variants, post-translational modifications, or small-molecule modulators—can lead to uncontrolled Ca²⁺ leak (defined as the inappropriate release of Ca²⁺ from the intracellular stores [14]), muscle weakness, hypercontracture, or malignant hyperthermia (MH) [15, 16]. Clinical reports have noted intriguing overlaps between statin intolerance and susceptibility to MH [17, 18], as well as the enrichment of RYR1 and CACNA1S variants in patients with severe statin-induced myopathy [19]. Animal models later confirmed increased SR Ca²⁺ leak and exaggerated contractility in RyR1 gain-of-function mutants treated with statins [20, 21].

Yet the central question remained: do statins directly bind RyR1? Or do they act indirectly through membrane perturbation, post-translational regulation, or altered mitochondrial metabolism?

The study by Molinarolo et al. [11] answers this definitively: atorvastatin binds directly to RyR1 at defined sites, induces large conformational transitions, and shifts the equilibrium toward the open, Ca²⁺-releasing state. Most remarkably, the authors show that not one but *three* atorvastatin molecules per RyR1 protomer are required for full activation—establishing a novel, multi-step drug-assembly mechanism that is unprecedented among known channel modulators.

## Triplet binding: a mechanism without precedent

The most striking discovery is the formation of a triplet of atorvastatin molecules within the pseudo-voltage-sensing domain (pVSD) of each RyR1 protomer [11]. Each statin binds in a hydrophobic cleft formed by helices S1, S3, and S4, making extensive interactions with both protein residues and neighboring statin molecules. The configuration is unexpectedly cooperative: hydrophobic moieties of one atorvastatin stabilize the binding of the others, creating a tightly packed assembly reminiscent of an engineered supramolecular complex rather than a typical ligand–receptor interaction.

This triplet binding configuration is directly linked to stepwise activation (Table 1):

**Table 1:** Structural interactions of Atorvastatin with RyR1 identified by Cryo-EM

Binding Site (RyR1 Domain)	Key Interacting Helices/Residues	Interaction Type	Functional Impact
Primary pocket near S4–S5 linker (“priming site”)	S4–S5 linker, S5′ helix	Hydrophobic contacts; minor steric displacement	Initiates small conformational shifts that “prime” the channel toward activation
Pseudo–voltage-sensing domain (pVSD) — Statin 1	S1, S3, S4 helices	Hydrophobic interactions	Establishes initial drug anchoring within the pVSD
pVSD — Statin 2	Adjacent S4 and S4–S5 linker residues	Cooperative hydrophobic packing with Statin 1	Drives outward displacement of S4, promoting pore dilation
pVSD — Statin 3	Hydrophobic cleft formed by S1/S3/S4	Inter-statin hydrophobic stabilization	Stabilizes the open conformation and maximizes Ca²⁺ leak
Putative ATP-binding site (weak density)	P-loop region near nucleotide-binding pocket	Weak occupancy; unclear interactions	Possible competition with ATP, requiring further investigation

First statin binding (“priming”): A single atorvastatin molecule binds to a site adjacent to the S4–S5 linker even when RyR1 is closed. This binding induces small but functionally meaningful rearrangements: a ~ 1.5 Å shift in the S4–S5 linker and S5’ helix, and subtle downward displacement of the large cytosolic cap. These structural shifts resemble the initial motions associated with “primed” gating states previously described in RyR1 cryo-EM studies.

Second and third statin binding (“opening”): Only in the open state can two additional molecules bind. Their insertion into the pVSD forces helices S4 and the S4–S5 linker outward by several Ångströms [11], triggering dilation of the pore and stabilization of the open conformation [13].

This sequential mechanism is reminiscent of the voltage-sensor activation in canonical voltage-gated channels [22], but here the pVSD is not inherently voltage sensitive. Instead, binding energy from multiple hydrophobic drug interactions drives the conformational changes that unleash Ca²⁺ flow.

## A molecular explanation for isoform specificity

One major clinical observation is that statins, despite widespread use, do not cause notable cardiac arrhythmias—implying that RyR2, the cardiac isoform [23–25], is relatively insensitive. There are critical amino acid differences between RyR1 and RyR2 directly within the atorvastatin binding pocket. Substitutions such as Phe4564→Met and Val4820→Phe introduce steric clashes that prevent atorvastatin engagement of RyR2 (Table 2). This structural explanation resolves long-standing clinical observations and underscores the skeletal muscle specificity of statin myotoxicity.

**Table 2:** Structural determinants of isoform specificity: RyR1 vs. RyR2 Atorvastatin binding

RyR1 Residue	RyR2 Corresponding Residue	Structural Effect of Substitution	Impact on Atorvastatin Binding
Phe4564	Met	Reduced aromatic surface; altered pocket shape	Prevents stable hydrophobic packing
Val4820	Phe	Increased steric bulk within pVSD	Steric clash blocks statin insertion
Leu4561	Ile	Minor change in side-chain orientation	Slight reduction in pocket compatibility
Ile4827	Met	Larger, more flexible side chain	Disrupts precise statin triplet positioning
Surrounding hydrophobic cleft residues	Multiple conservative substitutions	Overall decreased pocket hydrophobicity	Substantially lowers binding affinity

## Mechanistic insights into statin sensitivity in genetic myopathies

The investigations using the R615C RyR1 mutant (a substitution of arginine to cysteine at residue 615 that is the mouse ortholog of the human pathogenic R614C variant linked to MH [26]) are clinically relevant. This gain-of-function RyR1 channel is hypersensitive to activation, and exposure to atorvastatin shifts the open-channel probability far more markedly than in wild-type RyR1. Muscle biopsies from R614C carriers have shown severe contractures in response to statins [27]; the structural basis for this dangerous synergy has now been unveiled: the implication is profound: individuals with latent RyR1 gain-of-function variants may be particularly vulnerable to statin-induced hyperexcitability, Ca²⁺ overload, and myopathy.

## Unexpected observations at the ATP-binding site

Although less definitive, the detection of weak density at the ATP-binding pocket in the open-state structure suggests that atorvastatin might occasionally occupy this conserved regulatory site. Given that ATP physiologically stabilizes RyR1 opening [28], any competition or displacement could modulate channel behavior in vivo. More work is needed to determine whether this represents a true secondary binding site or incidental occupancy under high ligand concentrations.

## Statin chemistry and the path toward safer molecules

A comparison with the HMG-CoA reductase–atorvastatin complex offers immediate translational value. The hydrophilic dihydroxyheptanoic acid group (critical for enzymatic inhibition [29]) plays a minor role in RyR1 binding. Instead, the hydrophobic aromatic moieties (fluorophenyl, phenyl, and phenyl-carbamoyl groups) mediate the majority of interactions within the pVSD. Thus, modifying these hydrophobic groups could blunt RyR1 binding while preserving lipid-lowering efficacy. Medicinal chemists now have a clear structural roadmap for developing the first generation of “RyR1-silent” statins.

## Limitations and remaining questions

Despite the power of the structural insights, certain limitations merit careful consideration. Although necessary for visualization, the atorvastatin concentrations used exceed physiological levels. However, prior functional studies showing EC50 values around 300–400 nM suggest that the observed interactions are biologically relevant. RyR1 behavior in lipid environments or triadic junctions could differ; how phospholipid composition or accessory proteins (FKBP12, CaM, STAC3 [30]) modulate statin binding remains to be determined. These findings do not negate other proposed myopathic mechanisms; instead, they reveal a new central node within a broader pathological network. Functional validation directly linked to the cryo-EM constructs would strengthen causal claims. Statins vary in hydrophobicity and aromatic composition (Table 3); whether the triplet mechanism generalizes across the class will require additional structural work.

**Table 3:** Physicochemical features of major Statins and their predicted RyR1 interaction propensity

Statin	Hydrophobicity	Aromatic Moieties Present	Predicted RyR1 Interaction	Clinical Myopathy Incidence (Relative)
Atorvastatin	High	Fluorophenyl + phenyl + phenyl-carbamoyl	Strong; supports triplet binding	Moderate–high
Simvastatin	High	Single hydrophobic bicyclic moiety	Moderate; may partially fit pVSD cleft	Moderate
Lovastatin	High	Similar to simvastatin but less flexible	Moderate; possible partial binding	Moderate
Rosuvastatin	Low–moderate	Limited aromatic structure	Low; unlikely to support triplet assembly	Low
Pravastatin	Low	No major aromatic rings	Very low; minimal RyR1 affinity expected	Very low
Fluvastatin	Moderate	Biphenyl group	Moderate; possible single-site binding	Low–moderate
Pitavastatin	High	Multiple aromatic groups	Potentially moderate–high	Moderate

Even with all these caveats, the structural resolution of atorvastatin-RyR1 interactions represents a transformative leap forward.

## Clinical implications: a path toward precision Statin therapy

The translational implications are far-reaching. The enrichment of RYR1 and CACNA1S variants in statin-intolerant patients has been repeatedly observed but poorly integrated into clinical practice. This study provides the missing mechanistic link to justify (i) preemptive genetic screening in individuals with prior statin intolerance; (ii) screening in families with MH susceptibility; (iii) genotype-guided statin selection or dose adjustment.

The structural blueprint provided offers tangible opportunities for pharmaceutical innovation. Modifying specific aromatic moieties could create statins that maintain potency while eliminating RyR1 activation.

Patients with congenital myopathies or RyR1-related disorders may be uniquely susceptible to statin-induced exacerbations. The findings support heightened caution or alternative lipid-lowering strategies in these groups.

Statin myopathy can be thereby reframed as a receptor-mediated Ca²⁺ release disorder rather than a diffuse metabolic phenomenon. This conceptual clarity may enable the development of RyR1-stabilizing agents as protective co-therapies, improved biomarkers of early SR Ca²⁺ leak, and a shift from empiricism to mechanism in managing statin intolerance.

Statins are regarded as potentially myotoxic across the entire spectrum of neuromuscular disorders, not only in congenital myopathies related or unrelated to RyR1 variants. Individuals with pre-existing muscle disease may be predisposed to exacerbation of weakness, elevation of serum CK, or more severe muscle injury when exposed to statins. As a result, clinicians are advised to exercise heightened caution when prescribing these agents in patients with inherited or acquired neuromuscular conditions—such as muscular dystrophies, metabolic myopathies, inflammatory myopathies, and motor neuron disorders—by initiating at the lowest effective dose, closely monitoring clinical status and CK levels, and discontinuing therapy promptly if significant intolerance emerges. This broad precautionary stance underscores that statin-induced muscle toxicity is not confined to a narrow subset of genetic channelopathies but represents a wider clinical concern in vulnerable muscle phenotypes.

## Conclusions: a new era in Statin biology

Molinarolo et al. [11] deliver one of the most important mechanistic insights into statin toxicology since the discovery of HMG-CoA reductase inhibition. Their study provides definitive structural evidence that atorvastatin binds directly to RyR1 in a unique triplet assembly, sequentially activating the channel and promoting pathological Ca²⁺ leak. This work elegantly connects decades of clinical observations, pharmacogenomic associations, and physiological anomalies into a coherent molecular narrative. It opens the door to precision lipid therapy, rational statin redesign, and improved safety for millions of patients who rely on these cornerstone medications.

For the first time, the field has a detailed atomic map of how statins can trigger skeletal muscle dysfunction—and, importantly, a clear path toward eliminating this long-standing adverse effect.

## Data Availability

No datasets were generated or analysed during the current study.
